# PostOPbot: an artificial intelligence chatbot for postoperative care following tooth extraction

**DOI:** 10.1590/1807-3107bor-2026.vol40.089

**Published:** 2026-11-09

**Authors:** Aline Xavier FERRAZ, Pedro Felipe de Jesus FREITAS, Flavio Magno GONÇALVES, Michelle Nascimento MEGER, Guilherme Protzek GUARIZA, Rodrigo Nunes RACHED, Julia da Silva GERMINIANi, Allan ABUABARA, Angela Graciela Deliga SCHRODER, Flares BARATTO-FILHO, Bianca Marques de Mattos de ARAUJO, Cristiano Miranda de ARAUJO

**Affiliations:** (a) Universidade Tuiuti do Paraná – UTP – School of Health, Postgraduate Program in Human Communication Health, Curitiba, PR, Brazil.; (b) Universidade Tuiuti do Paraná – UTP – School of Dentistry, Department of Endodontics, Orofacial Pain and Temporomandibular Disorders, Curitiba, PR, Brazil.; (c) Universidade da Região de Joinville – Univille – School of Medicine, Department of Dentistry, Joinville, SC, Brazil.; (d) Pontifícia Universidade Católica do Paraná – PUCPR – School of Medicine and Life Sciences, Postgraduate Program in Dentistry, Curitiba, PR, Brazil.

**Keywords:** Tooth Extraction, Postoperative Period, Postoperative Care, Generative Artificial Intelligence, Artificial Intelligence, Intelligent Systems

## Abstract

Postoperative care following dental extractions requires clear and continuous guidance; however, patient adherence to postoperative instructions remains suboptimal, and access to timely professional support may be limited. This study aimed to develop and evaluate PostOPbot, an artificial intelligence (AI)-based virtual assistant designed to provide standardized postoperative guidance through natural language processing. The system was implemented in Python with a web-based interface and integrated with the ChatGPT-4o application programming interface through a conversational endpoint. Postoperative recommendations were identified through a structured PubMed search and incorporated into a static prompt that defined the system’s operational rules, restricted its scope to postoperative counseling, and guided the interpretation of user input. The rule set was embedded into the code so that each user query was combined with predefined instructions before being processed by the language model. The chatbot was evaluated using three simulated postoperative scenarios representing different time intervals after tooth extraction. For each scenario, the system was expected to provide a predefined set of recommendations, and its performance was assessed by determining whether each item was delivered spontaneously or only after explicit prompting. PostOPbot provided most expected recommendations with few omissions, and missing recommendations were consistently generated upon direct request. Although large language model-based systems have inherent limitations, the findings suggest that PostOPbot may serve as a complementary AI tool to reinforce postoperative guidance without replacing professional evaluation.

## Introduction

Postoperative care in dentistry is essential to ensure appropriate recovery and prevent complications, playing a decisive role in the success of surgical procedures.^1^ These measures include guidance on oral hygiene, diet, appropriate medication use, attendance at follow-up appointments, and, importantly, the early recognition of warning signs such as severe pain, bleeding, marked swelling, or infection.^2,3^

Despite the well-established importance of these recommendations, patient adherence to postoperative instructions remains suboptimal. Several factors contribute to this problem, including communication gaps between clinicians and patients, limited health literacy, anxiety, fear, and misunderstandings about the healing process^4,5^. Poor adherence may result in complications such as alveolar osteitis, infection, excessive bleeding, persistent pain, injury to adjacent structures, and impaired wound healing, thereby increasing treatment costs and the need for additional clinical interventions.^2,3^

These challenges underscore the importance of close and continuous postoperative follow-up supported by clear, accessible, and timely information.^4,5^ However, in high-demand clinical settings, dentists may not always be available to respond promptly to patients’ questions and concerns during recovery.^2,3^

In response to this challenge, recent advances in artificial intelligence (AI), particularly natural language processing (NLP), have emerged as promising tools for enhancing communication between healthcare providers and patients.^6-8^ NLP uses algorithms capable of understanding, interpreting, and generating human language, enabling the development of interactive systems such as chatbots. These systems provide rapid, standardized, and continuous support, thereby improving access to reliable information. Although tools such as ChatGPT-4o can provide general recommendations and facilitate broader patient communication, specialized chatbots developed for dental clinical applications are more likely to deliver accurate, context-specific interactions tailored to patients’ needs and the characteristics of the procedures performed.^9^ In medicine, these technologies have demonstrated effectiveness across a range of applications, including triage, remote monitoring, diagnostic support, and the promotion of self-care.^10-16^ However, their application in dentistry, particularly for postoperative follow-up, remains limited. To date, no published studies have described AI-based interactive chatbots designed to support patients after dental procedures, highlighting an important gap and an opportunity for innovation.

Against this background, exploring digital strategies that incorporate this technology may improve patient engagement with postoperative care and reduce the risk of complications. Therefore, the present study aimed to develop and preliminarily evaluate a natural language processing–based virtual assistant designed to automate postoperative support for patients undergoing tooth extractions using simulated postoperative scenarios.

## Methods

### Study design

This study consisted of a simulation-based performance assessment in which predefined postoperative scenarios were used to evaluate the chatbot’s responses under controlled conditions without real patient interaction.

### Chatbot overview

Interactive and context-aware communication was enabled by developing the chatbot in the Python programming language using the PyCharm integrated development environment (version 2023.2.3). Integration with the ChatGPT-4o application programming interface (API) was implemented using the *openai* library.

The chatbot was developed according to the following principles:

- **Focus on postoperative follow-up**: The chatbot, named PostOPbot (Post Extraction Support Tool), was designed exclusively to provide information related to the postoperative period following dental extractions. Intended to complement clinical care, PostOPbot provides guidance on immediate and late postoperative measures while addressing common questions regarding warning signs and potential complications.
- **Interactive natural language-based approach**: PostOPbot uses NLP techniques to support dynamic, user-adapted interactions in more than 50 languages. The system conducts context-appropriate dialogue and, when necessary, asks targeted questions to facilitate the delivery of appropriate postoperative guidance.

The development workflow of PostOPbot is illustrated in Figure 1.

**Figure 1:**
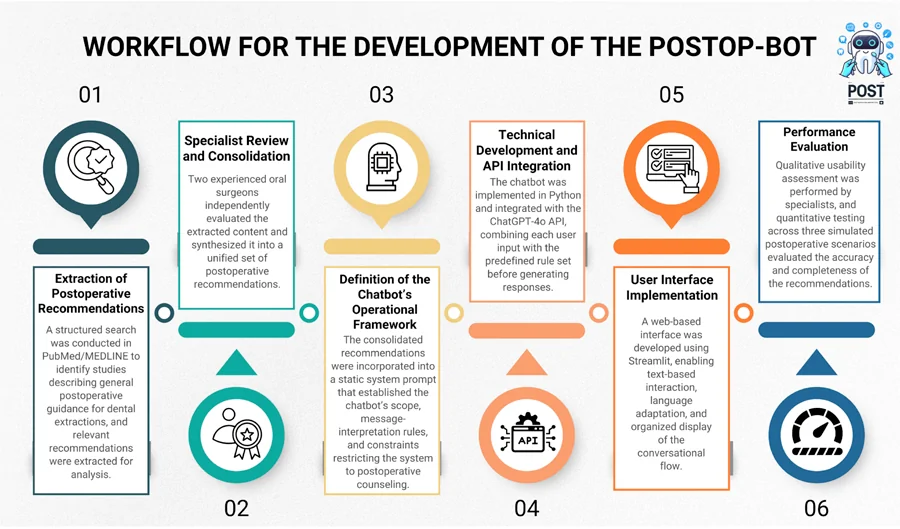
Development workflow of PostOPbot.

### Information sources

The search for information sources to support the chatbot’s responses was conducted in two stages. The first stage involved synthesizing general postoperative recommendations for dental extractions identified in the scientific literature, whereas the second consisted of expert review by experienced oral surgeons.

In the first stage, content selection was performed by two independent reviewers. Screening was conducted on the Rayyan platform (https://www.rayyan.ai/) to ensure an independent, blinded review process, and disagreements were resolved by a third reviewer. The literature search was performed in PubMed/MEDLINE using the descriptors “Tooth Extraction” and “Postoperative Care.” Article titles and abstracts were screened for relevance, and the full text was assessed when an article was considered potentially eligible.

The eligibility criteria were as follows: (1) provision of general postoperative guidance related to care after dental extractions and (2) exclusion of studies addressing medication-based postoperative management. General postoperative recommendations were extracted from each included study. The final selection resulted in a standardized set of postoperative recommendations covering immediate and late postoperative care, oral hygiene, dietary guidance, activity restrictions, and warning signs.

In the second stage, two oral surgeons with more than 10 years of clinical experience, who had not participated in either the literature search or chatbot development, evaluated the relevance of the extracted recommendations and synthesized them into a unified set of postoperative recommendations. This consolidated set of recommendations was subsequently incorporated into the chatbot as the basis for response generation, ensuring consistency with postoperative guidance reported in the literature and routinely applied in clinical practice.

### Development of the chatbot and conversational logic

PostOPbot was developed as a rule-based conversational system integrated with the ChatGPT-4o language model through its API. Clinical recommendations synthesized from the literature review were incorporated into the system as a static initial prompt defining the chatbot’s operational scope and establishing the parameters governing message interpretation and response generation. This instruction set restricts the system to postoperative counseling related to dental extractions, standardizes the terminology used, and prevents the generation of diagnoses, prescriptions, or guidance beyond the chatbot’s intended clinical scope.

Communication with the language model occurred sequentially through the *chat completion* mechanism. At each interaction, the user’s input was combined with the predefined rule set and forwarded to the model for response generation. This process preserved thematic continuity and ensured that all responses remained aligned with the predefined instructions. The conversation history was stored internally within the application, allowing contextual information to be retrieved and updated throughout the dialogue.

Message interpretation—including language recognition, understanding of postoperative reports, and identification of patient-reported symptoms—was performed by the language model. The integrated rule layer served as an additional control mechanism, filtering and refining responses to ensure alignment with the established clinical scope. This architecture promoted consistency, reproducibility, and adherence to the incorporated clinical recommendations.

The programming code used to develop PostOPbot is available as open-access material (DOI: 10.5281/zenodo.17840660).

### Interface development

The PostOPbot user interface was implemented as a web application using the Streamlit framework (Figure 2). The interface enables direct text-based interaction and presents an introductory message guiding users on how to begin the conversation. Visual elements, including header images and a sidebar containing bibliographic references, support usability and provide context for the tool’s purpose.

**Figure 2:**
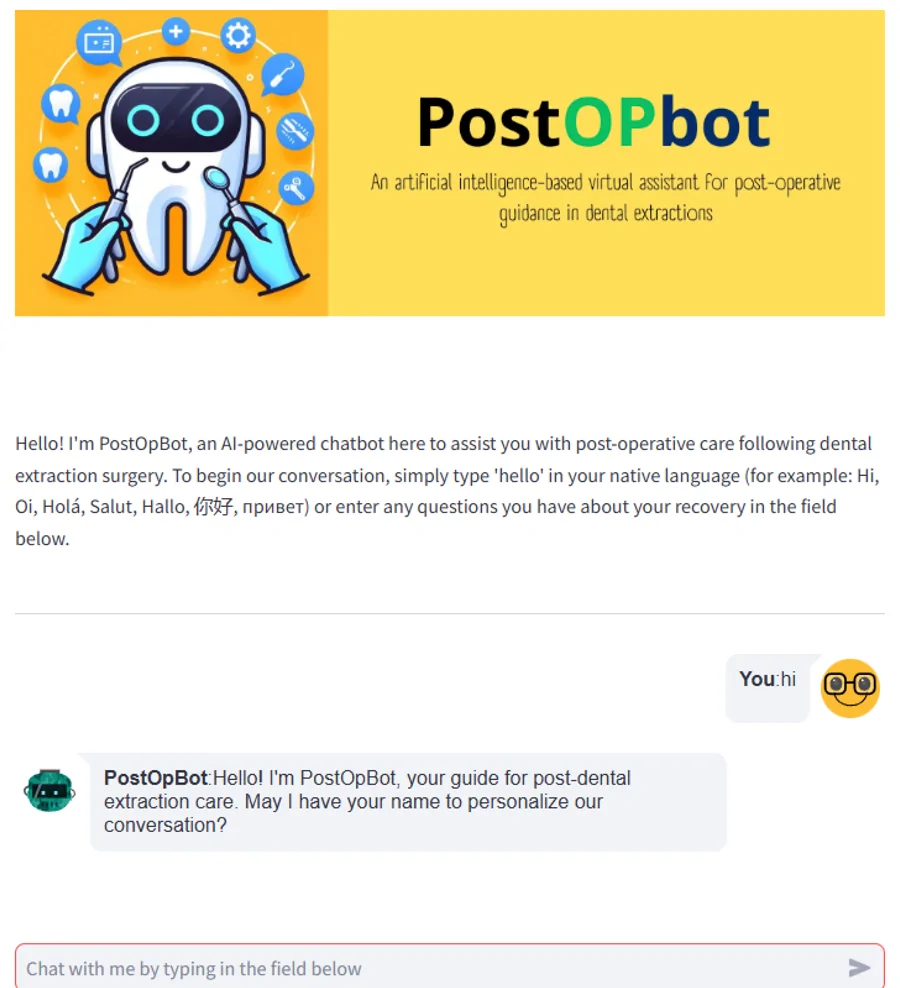
User interface of PostOPbot, illustrating the conversational layout and interaction between the user and the chatbot.

User input is entered through a dedicated text field, which initiates message exchange with the language model. The interface displays the conversation in an organized format that distinguishes user messages from chatbot responses, promoting clarity and facilitating navigation throughout the interaction. A scrolling mechanism automatically displays the most recent messages, ensuring continuity and readability during extended conversations.

The system also supports automatic language adaptation, allowing the chatbot to respond in the same language used by the user at the beginning of the interaction. PostOPbot is accessible on desktop and mobile devices at https://postopbot.streamlit.app/.

### Performance evaluation

The chatbot’s performance was evaluated using qualitative and quantitative approaches. The qualitative assessment focused on usability, whereas the quantitative assessment evaluated the accuracy of the postoperative recommendations provided.

### Qualitative evaluation

The qualitative evaluation of PostOPbot was performed by four dentists experienced in tooth extraction procedures. Although they were members of the research team, these professionals did not participate in either the information selection stage or chatbot development, thereby ensuring an impartial evaluation.

The qualitative assessment addressed the following aspects:

- **Usability**: evaluators examined ease of use, clarity of the information presented, and interface navigability during interaction.
- **Suggestions for improvement**: evaluators provided recommendations for system enhancement based on their clinical experience and direct interaction with the chatbot.

### Quantitative evaluation

The quantitative analysis was based on the chatbot’s performance in three simulated clinical scenarios representing different postoperative time points. These scenarios reproduced situations commonly experienced by patients following tooth extraction and were designed to evaluate the appropriateness of the chatbot’s responses according to the elapsed postoperative period. Each scenario included a predefined set of expected recommendations.

- Scenario 1: Tooth extraction performed 5 minutes earlier.
- Scenario 2: Tooth extraction performed 60 minutes earlier.
- Scenario 3: Tooth extraction performed more than 49 hours earlier.

Each scenario represented a distinct phase of the postoperative period. The first two scenarios (5 and 60 minutes) focused on immediate postoperative care, including bleeding control, protection of the surgical site, and activity restrictions. The third scenario (49 hours) represented the post-acute phase, in which oral hygiene measures, gradual resumption of activities, and monitoring of healing become more relevant. This time point was selected because it immediately follows the 48-hour postoperative threshold, marking the transition between early and late postoperative care in several clinical recommendations, particularly those applicable only after this interval. This design enabled the evaluation of the chatbot’s ability to adapt its recommendations according to postoperative progression.

The following variables were analyzed:

- **Percentage of correct recommendations**: for each scenario, the proportion of clinically appropriate responses was calculated according to predefined criteria. Each chatbot response was systematically compared with a predefined checklist of expected postoperative recommendations. Evaluators determined whether each recommendation was explicitly included in the response, accepting wording differences when the clinical meaning was preserved. Responses were classified as correct only when they contained the complete set of expected recommendations; incomplete or discordant responses were classified as incorrect.
- **Number of spontaneously provided recommendations**: this variable represented the number of recommendations generated spontaneously by the chatbot in each scenario relative to the expected set. The percentage of recommendations provided was calculated based on the total expected for each scenario. When a recommendation was omitted, the evaluator explicitly requested that recommendation to determine whether the chatbot could provide it when prompted.

A binary classification approach was adopted to ensure an objective and reproducible assessment by focusing exclusively on the presence or absence of clinically essential information. This approach was considered appropriate because omission of key postoperative recommendations may have greater clinical relevance than differences in wording. Chatbot responses were evaluated independently by two evaluators with clinical experience in oral surgery. Before the main analysis, both inter- and intra-rater agreement were assessed using Cohen’s kappa coefficient, demonstrating excellent reliability (κ > 0.90). Discrepancies were resolved by consensus.

### Sample size calculation

The minimum sample size was determined from the results of a pilot study. A conservative estimate was obtained by using the parameter with the lowest observed value in the pilot study, namely the proportion of spontaneously provided recommendations (90%), for the sample size calculation. Because no finite population was defined, an infinite-population assumption was adopted to estimate the minimum number of interactions required to ensure stable performance estimates. Assuming a 5% margin of error and a 95% confidence level, the minimum required sample size was estimated at 140 interactions.

## Results

### Sources of information

A total of 249 references were identified through the PubMed/MEDLINE search using the predefined descriptors. After screening the titles and abstracts, 12 references were selected for full-text review, five of which were excluded because they focused solely on medication-based postoperative care. Consequently, seven studies^1,2,17-21^ were included, and general postoperative recommendations were extracted from each (Table 1). Following expert review, the recommendations were consolidated and are presented in Table 2.

**Table 1:** General postoperative recommendations extracted from each included study.

Author	General postoperative recommendations
Shenoi RS et al., 2021^26^	a) Gauze/bleeding: keep the folded gauze pressed for 30 to 45 minutes after the extraction.
	b) Do not spit: avoid spitting during the first 24 hours after the extraction.
	c) Straws: do not use straws.
	d) Diet: consume a soft, semi-liquid diet at a cool or lukewarm temperature.
	e) Ice: apply ice to the outside of the face in the area where the extraction was performed.
	f) Rinsing: rinse with warm water starting 48 hours after the extraction.
	g) Mouth-opening exercises: perform mouth-opening exercises starting 48 hours after the extraction.
	h) Oral hygiene: maintain proper oral hygiene.
	i) Physical activity: avoid strenuous exercise for one week after the extraction.
	j) Habits to avoid:
	avoid smoking or using tobacco during the postoperative period.
	do not consume alcoholic or carbonated beverages after the extraction.
Alvira-González et al., 2015^14^	I. Immediate care (first 24 hours)
	a) Gauze: keep the folded gauze pressed for 30 minutes after the extraction.
	b) Liquids/rinsing: avoid rinsing with any liquid during the first 24 hours.
	c) Spitting: do not spit. If the saliva is slightly blood-tinged, it should be swallowed.
	d) Ice: apply ice wrapped in a cloth to the outside of the face where the extraction was performed. Ice should be used during the first 24 hours to help reduce swelling and discomfort.
	e) Sleeping position: use two pillows to sleep, keeping the head slightly elevated.
	f) Physical activity/exercise: do not perform work or exercise that requires significant exertion.
	II. Diet and Habits
	a) Diet: consume a soft or semi-liquid diet. Food temperature should be cool or lukewarm.
	b) Hydration: drink plenty of fluids.
	c) Foods to avoid: avoid hot and irritating foods.
	d) Chewing: chew on the opposite side.
	e) Tobacco: avoid smoking during the postoperative period (7 days after surgery).
	f) Beverages: do not consume alcoholic or carbonated drinks during the week following surgery.
	III. Hygiene and healing
	a) General hygiene: maintain proper oral hygiene.
	b) Brushing: brush your teeth as usual, three times a day.
	c) Brushing the surgical site: brush the surgical area using a surgical toothbrush after each meal.
	d) Antiseptic rinses: starting one day after surgery, and for 15 days, rinse with the recommended mouthwash.
	IV. Management of complications
	a) Persistent Bleeding: If heavy bleeding occurs, remove the excess blood with gauze, place a new gauze pad on the bleeding site (do not use cotton), and press it firmly with your teeth for 30 minutes (repeat 2–3 times). If bleeding continues, contact your surgeon.
	b) Swelling (edema): facial swelling usually appears several hours after surgery and may increase during the first 2 to 3 postoperative days.
	c) Trismus (difficulty opening the mouth): trismus is a common complication that gradually improves. Do not attempt to force mouth opening.
	d) Pain: postoperative pain may last several days (4–5 days), often worsening at night and after meals. Take all medications as prescribed to relieve discomfort. If the pain becomes severe, contact your surgeon.
Demyati et al., 2025^15^	I. Immediate care and bleeding
	a) Gauze: follow the recommendation to bite on the gauze for 30 minutes until the bleeding stops.
	b) Do not bite: follow the recommendation not to bite your cheeks or lips.
	c) Spitting/saliva: follow the recommendation not to spit and to swallow your saliva even if it is blood-tinged.
	d) Initial hygiene: follow the recommendation not to rinse or brush the extraction site within the first 24 hours.
	e) Anesthesia: follow the recommendation not to drink or eat anything until the effect of the anesthesia has worn off.
	II. swelling control and positioning
	a) Ice: apply an ice pack during the first 20 minutes after the extraction.
	b) Sleep: use the double-pillow technique when sleeping to help reduce bleeding.
	III. Diet and oral habits
	a) Diet: follow the recommendation to maintain a cold and soft diet during the first 24 hours after the procedure.
	b) Straws: follow the recommendation not to use a straw for a few days after surgery.
	c) Smoking: if applicable, follow the recommendation not to smoke during the first 72 hours after the procedure.
	IV. Later hygiene and healing
	a) Brushing (after 24 h): resume gentle brushing and rinsing one day after surgery, once the bleeding has stopped.
	b) Continuous hygiene: maintain dental hygiene and perform gentle brushing regularly after the day of extraction.
	c) Rinsing: use warm salt water frequently after meals.
	V. Physical activity and medication
	a) Exercise: avoid strenuous physical activity.
	b) Medication (general): follow all medication instructions exactly as prescribed by the surgeon.
Sybil D et al., 2022^27^	I. Immediate care and bleeding
	Cotton pad: bite on the cotton pad for about one hour.
	Ice: use ice packs on the cheek (externally) during the first 24 hours to reduce swelling. The instruction is to remove the ice one hour after the procedure.
	No rinsing/spitting: do not spit or rinse for at least 24 hours.
	II. Diet and eating
	Temperature and consistency: consume semisolid and cold (or soft) foods during the first 24 hours.
	Foods to avoid: do not consume hot, solid, or spicy foods during the first 24 hours.
	Chewing: chew only on the side opposite the extraction site for 2 to 3 days.
	Straws: do not use a straw to drink liquids for 24 hours.
	Anesthesia: do not eat or drink anything until the effect of the anesthesia has worn off.
	III. Oral hygiene (after 24 Hours)
	Saline rinses: after 24 hours, begin rinsing with warm salt water 5 to 7 times a day.
	Brushing: brush regularly.
	IV. Medication and Pain
	Take medication: take all prescribed medications (antibiotics and pain relievers) on time to prevent pain or discomfort at the extraction site.
	Adherence: follow the full prescribed course of antibiotics and pain relievers exactly as directed.
	V. Habits to avoid
	Smoking and alcohol: avoid smoking, pan masala, tobacco, and alcohol. Smoking restriction is strongly recommended.
Postoperative Instructions for Extractions/Oral Surgery – Pediatric Dentistry^18^	I. Immediate care and bleeding
	a) Numbness: the mouth will remain numb for approximately two to four hours. It is important to monitor the child to ensure they do not bite, scratch, or injure the cheek, lips, or tongue during this period.
	b) Bleeding: occasional leakage may occur (pink or blood-tinged saliva).
	c) Gauze: hold the gauze with firm pressure against the surgical site until the leakage stops. It may be necessary to replace the gauze or repeat this step.
	d) Persistent bleeding: if bleeding continues for more than two hours, contact the office.
	II. Surgical site care (day of surgery)
	a) Do not disturb: do not disturb the surgical area today.
	b) Movements: do not stretch the lips or cheeks to look at the area.
	c) Hygiene: do not rinse vigorously, use mouthwash, or probe the area with fingers or other objects.
	III. Activities, diet, and habits
	a) Exercise: avoid physical activity and exertion on the day of surgery. Return to normal activities as tolerated.
	b) Smoking: smoking can delay healing after oral surgery.
	c) Diet: sfter bleeding has stopped, the patient may drink cold, noncarbonated liquids, but should NOT use a straw.
	d) Hydration: encourage fluid intake to prevent dehydration.
	e) Foods (first day): cold and soft foods such as ice cream, gelatin, pudding, and yogurt are ideal on the first day.
	f) Diet progression: food consistency may progress as tolerated beginning on the second day.
	g) Foods to avoid: avoid foods such as nuts, sunflower seeds, and popcorn until healing is better established, as they can become lodged in surgical areas.
	IV. Oral hygiene
	a) Brushing (today): Teeth may be brushed and flossed gently today, but the surgical site should be avoided.
	b) Cleaning: make every effort to clean the teeth within comfort limits, even if pain and swelling prevent vigorous brushing in all areas.
	c) Rinsing (starting tomorrow): beginning tomorrow, warm salt-water rinses (½ teaspoon of salt in one cup of water) may be used after meals.
	V. Swelling and temperature
	a) Ice (first 24 Hours): If swelling occurs, ice packs may be used during the first 24 hours (10 minutes on, then 10 minutes off) to reduce swelling and bruising.
	b) Warm/moist compresses (after 24 hours): If swelling persists after 24 hours, warm moist compresses (10 minutes on, then 10 minutes off) may help.
	c) Late swelling: if swelling occurs after 48 hours, contact the office.
	d) Fever: a mild fever (up to 38 °C or 100.5 °F) is not uncommon during the first 48 hours. If the fever is higher or persistent, contact the office.
	VI. Other complications and follow-up
	a) Sutures: sutures help control bleeding and support healing. If sutures come out within the first 48 hours, call the office.
	b) Dry socket: be alert for dry socket, which typically occurs between the third and fifth days after extraction of a permanent tooth and is characterized by persistent throbbing pain in the jaw. If this occurs, call the office.
	c) Follow-up: schedule the next appointment.
Postoperative Instructions for Extractions/Oral Surgery - American Academy of Pediatric Dentistry, 201617	I. Immediate care and bleeding
	a) Numbness: the mouth will remain numb for approximately two to four hours. It is important to monitor the child to ensure they do not bite, scratch, or injure the cheek, lips, or tongue during this period.
	b) Bleeding: occasional leakage may occur (pink or blood-tinged saliva).
	c) Gauze: hold the gauze with firm pressure against the surgical site until the leakage stops. It may be necessary to replace the gauze or repeat this step.
	d) Persistent bleeding: if bleeding continues for more than two hours, contact the office.
	II. Surgical site care (day of surgery)
	a) Do not disturb: do not disturb the surgical area today.
	b) Movements: do not stretch the lips or cheeks to look at the area.
	c) Hygiene: do not rinse vigorously, use mouthwash, or probe the area with fingers or other objects.
	III. Activities, diet, and habits
	a) Exercise: avoid physical activity and exertion on the day of surgery. Return to normal activities as tolerated.
	b) Smoking: smoking can delay healing after oral surgery.
	c) Diet: after bleeding has stopped, the patient may drink cold, noncarbonated liquids, but should NOT use a straw.
	d) Hydration: encourage fluid intake to prevent dehydration.
	e) Foods (first day): cold and soft foods such as ice cream, gelatin, pudding, and yogurt are ideal on the first day.
	f) Diet progression: food consistency may progress as tolerated beginning on the second day.
	g) Foods to avoid: Avoid foods such as nuts, sunflower seeds, and popcorn until healing is better established, as they can become lodged in surgical areas.
	IV. Oral Hygiene
	a) Brushing (today): Teeth may be brushed and flossed gently today, but the surgical site should be avoided.
	b) Cleaning: make every effort to clean the teeth within comfort limits, even if pain and swelling prevent vigorous brushing in all areas.
	c) Rinsing (starting tomorrow): Beginning tomorrow, warm salt-water rinses (½ teaspoon of salt in one cup of water) may be used after meals.
	V. Swelling and temperature
	a) Ice (first 24 hours): if swelling occurs, ice packs may be used during the first 24 hours (10 minutes on, then 10 minutes off) to reduce swelling and bruising.
	b) Warm/moist compresses (after 24 hours): if swelling persists after 24 hours, warm moist compresses (10 minutes on, then 10 minutes off) may help.
	c) Late swelling: if swelling occurs after 48 hours, contact the office.
	d) Fever: a mild fever (up to 38 °C or 100.5 °F) is not uncommon during the first 48 hours. If the fever is higher or persistent, contact the office.
	VI. Other complications and follow-up
	a) Sutures: sutures help control bleeding and support healing. If sutures come out within the first 48 hours, call the office.
	b) Dry socket: be alert for dry socket, which typically occurs between the third and fifth days after extraction of a permanent tooth and is characterized by persistent throbbing pain in the jaw. If this occurs, call the office.
	c) Follow-up: schedule the next appointment.
Aloy-Prósper et al., 2020^28^	I. Immediate care and bleeding
	a) Gauze: keep the folded gauze pressed for 30 minutes after the extraction.
	b) Do not spit: do not spit. Slightly blood-tinged saliva that may occur for several hours should be swallowed.
	c) Heavy bleeding: if heavy bleeding occurs, remove the excess blood with gauze, place a new gauze pad over the bleeding site (do not use cotton), and press it firmly with your teeth for 30 minutes. Repeat this process 2 to 3 times. If bleeding continues, contact the surgeon.
	d) Initial rinsing: avoid rinsing with any liquid during the first 24 hours to prevent dislodging the blood clot.
	II. Swelling (edema) and pain control
	a) Ice (cold): apply ice wrapped in a cloth to the outside of the face where the extraction was performed. Ice should be applied during the first 24 hours to help reduce inflammation and discomfort.
	b) Analgesics and anti-inflammatories: take pain relievers and anti-inflammatory medications as instructed by the surgeon, following the prescribed schedule.
	III. Hygiene and infection prevention
	a) General hygiene: maintain adequate oral hygiene.
	b) Brushing: brush your teeth as usual, three times a day.
	c) Brushing the surgical site: brush the surgical site using a surgical toothbrush after each meal. Brush this area very gently.
	d) Sutures: keep sutures clean by brushing them gently.
	e) Chlorhexidine rinses: rinse with 0.12% chlorhexidine starting 24 hours after surgery (three times a day for ten days).
	f) Antibiotics: take antibiotics according to the surgeon’s instructions and prescribed schedule.
	g) Risk Factor: poor hygiene and failure to perform chlorhexidine rinses were identified as major points of noncompliance with treatment.
	IV. Diet and Habits
	a) Diet: consume a soft or semi-liquid diet, opting for cool or lukewarm foods.
	b) Chewing: chew on the opposite side.
	c) Foods to avoid: avoid hot and irritating foods.
	d) Physical activity: do not perform work or exercise that requires significant exertion.
	e) Smoking: avoid smoking during the postoperative period (seven days after surgery), since it increases infection rates and delays healing. Smoking was identified as one of the major noncompliance factors.
	f) Beverages: do not consume alcoholic beverages or soft drinks during the week after surgery.
	g) Carbonated drinks: do not drink carbonated beverages because the blood clot may weaken and dislodge.
	V. Information on complications
	The article also provided additional information on expected complications:
	Facial edema: facial swelling typically appears several hours after surgery and increases during the first two to three postoperative days.
	Pain: postoperative pain may last several days (four to five days), often worsening at night and after meals.
	Trismus: trismus (difficulty opening the mouth) is a common complication after wisdom tooth extraction and gradually resolves over time.

**Table 2:** Scenarios and corresponding postoperative recommendations.

Scenario	Recommendations
Scenario 1 – 5 minutes after extraction (14 recommendations)	1. Apply folded gauze with pressure for 30 minutes after tooth extraction.
	2. Avoid spitting for the first 24 hours.
	3. Avoid rinsing with any liquids for the first 24 hours.
	4. Avoid suction; do not use straws.
	5. Consume a soft or semi-liquid diet, opting for cool or lukewarm foods.
	6. Apply ice wrapped in a cloth to the outside of the face for the first 24 hours.
	7. Maintain proper oral hygiene (clean the surgical site very gently).
	8. Avoid smoking for 7 days and refrain from consuming alcoholic beverages during the following week.
	9. Avoid carbonated drinks.
	10. Take antibiotics, pain relievers, and anti-inflammatories only as prescribed.
	11. Rinse with warm water after 48 hours; avoid vigorous rinsing.
	12. Use 0.12% chlorhexidine mouthwash starting 24 hours after surgery, as directed.
	13. Perform mouth-opening exercises after 48 hours.
	14. Avoid strenuous physical activity for 7 days.
Scenario 2 – 60 minutes after extraction (13 recommendations)	1. Avoid spitting for the first 24 hours.
	2. Avoid rinsing with any liquids for the first 24 hours.
	3. Avoid suction; do not use straws.
	4. Consume a soft or semi-liquid diet, opting for cool or lukewarm foods.
	5. Apply ice wrapped in a cloth to the outside of the face for the first 24 hours.
	6. Maintain proper oral hygiene (clean the surgical site very gently).
	7. Avoid smoking for 7 days and refrain from consuming alcoholic beverages during the following week.
	8. Avoid carbonated drinks.
	9. Take antibiotics, pain relievers, and anti-inflammatories only as prescribed.
	10. Rinse with warm water after 48 hours; avoid vigorous rinsing.
	11. Use 0.12% chlorhexidine mouthwash starting 24 hours after surgery, as directed.
	12. Perform mouth-opening exercises after 48 hours.
	13. Avoid strenuous physical activity for 7 days.
Scenario 3 – 49 hours after extraction (10 recommendations)	1. Avoid suction; do not use straws.
	2. Consume a soft or semi-liquid diet, opting for cool or lukewarm foods.
	3. Maintain proper oral hygiene (clean the surgical site very gently).
	4. Avoid smoking for 7 days and refrain from consuming alcoholic beverages during the following week.
	5. Avoid carbonated drinks.
	6. Take antibiotics, pain relievers, and anti-inflammatories only as prescribed.
	7. Rinse with warm water after 48 hours; avoid vigorous rinsing.
	8. Use 0.12% chlorhexidine mouthwash starting 24 hours after surgery, as directed.
	9. Perform mouth-opening exercises after 48 hours.
	10. Avoid strenuous physical activity for 7 days.

### Qualitative evaluation

All evaluators considered the chatbot easy to use, and particularly highlighted its ability to communicate effectively in multiple languages. However, they suggested improvements to ensure that the chatbot provided more detailed guidance following tooth extraction, explicitly emphasizing that the information was limited to initial postoperative instructions and that professional evaluation may still be necessary. They also recommended presenting each recommendation separately to minimize potential misunderstandings.

### Quantitative evaluation

The percentage of correct recommendations generated by PostOPbot was evaluated across three simulated clinical scenarios representing different postoperative time points following tooth extraction: 5 minutes, 60 minutes, and 49 hours. The chatbot was expected to provide 14, 13, and 10 recommendations for the respective scenarios.

Overall, most recommendations achieved 100% correctness across all scenarios. Variations were observed primarily for recommendations R4 and R9, with accuracy below 90% in one scenario. All responses classified as incorrect resulted from the partial omission of the expected postoperative recommendation. Figure 3 presents the percentage of correct (“Yes”) recommendations generated by the chatbot for each item according to the postoperative time point.

**Figure 3:**
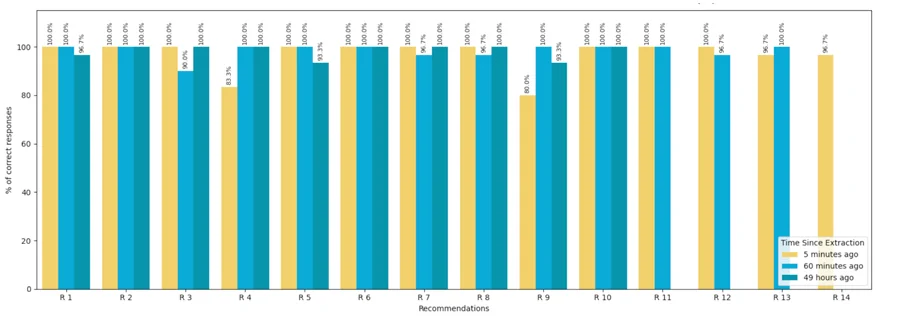
Percentage of correct responses for each postoperative recommendation across the three simulated scenarios.

Across all evaluated scenarios, the mean number of recommendations not provided spontaneously by the chatbot remained below 1.0. The highest frequency of omissions occurred in the 5-minute scenario, with a mean of 0.40 ± 0.56 recommendations omitted. In the 60-minute and 49-hour scenarios, the corresponding values were 0.20 ± 0.76 and 0.17 ± 0.37, respectively. Whenever a recommendation was omitted from the initial response, the evaluator explicitly requested that recommendation from the chatbot to determine whether it could provide the expected postoperative guidance when prompted. In every instance, PostOPbot subsequently generated the requested postoperative guidance, demonstrating its ability to respond appropriately following explicit prompting.

## Discussion

Postoperative follow-up remains one of the principal clinical challenges in dental practice. Although diagnosis and treatment planning are well established in most cases, more complex situations may require a differentiated diagnostic approach and closer patient monitoring.^2,4,19^ The present study developed and preliminarily evaluated PostOPbot, a natural language processing–based virtual assistant designed to automate postoperative follow-up for patients undergoing tooth extraction, while providing initial support for clinical reasoning. The system demonstrated consistent performance, with a high proportion of correct recommendations, clear guidance, and effective user interaction. No unsafe or clinically inappropriate recommendations were identified during the evaluation. All incorrect responses resulted from partial omission of expected information rather than from the generation of misleading or potentially harmful guidance.

Adherence to health recommendations is frequently suboptimal and remains a global challenge in clinical practice. This level of adherence varies across populations and treatment modalities, reflecting persistent limitations in patients’ implementation of the recommendations they receive.^2^ Sybil et al.^19^ evaluated postoperative adherence using a mobile application and observed better adherence when recommendations were delivered digitally rather than verbally or in written form. The authors attributed this finding to reduced language barriers, lower postoperative stress, and a lower likelihood of forgetting or misunderstanding the instructions received. Building on these findings, the present study developed a digital tool designed to provide standardized and accessible postoperative guidance as a complement to conventional patient instructions. Although not intended to replace professional follow-up, such a system may provide additional support throughout the postoperative period.

NLP, a subfield of AI, focuses on enabling computers to understand and generate human language.^22^ NLP-based chatbots have shown considerable potential for patient communication by interpreting natural-language queries and providing clear, continuous guidance. Because many individuals seek health information online, reliable systems may reduce exposure to inaccurate content while offering immediate support when direct professional consultation is unavailable.^23^ Advances in large language models (LLMs) have further expanded these capabilities, with studies suggesting their usefulness in supporting clinical reasoning,^24^ although concerns regarding hallucinations remain.^7,25^ Within this context, a chatbot developed for postoperative follow-up may strengthen clinical communication by providing standardized recommendations that are continuously available, multilingual, and easily accessible, thereby improving patient understanding and supporting self-management during recovery. From the patient’s perspective, such tools may represent an accessible source of immediate postoperative information that can help clarify patients’ questions, reinforce recommendations provided during the clinical appointment, and reduce uncertainty throughout recovery. Nevertheless, these systems should be used exclusively as complementary resources, and patients should be clearly informed that these systems do not replace professional evaluation, particularly when warning signs or complications arise. Patients should also be made aware of the role and limitations of AI in generating the information presented.^26^

LLMs have demonstrated meaningful performance in tasks related to oral and maxillofacial surgery and are increasingly being incorporated into education, clinical support, research, and patient education.^27^ In theoretical assessments, GPT-4 achieved 76.8% accuracy, outperforming the other evaluated models, although performance across the evaluated LLMs was lower in areas such as pharmacology.^28^ Similar findings have been reported in board-style examinations, in which GPT-4o achieved 83.7% accuracy; however, these results remain insufficient to support its independent clinical use.^29^ Narrative reviews further indicate that LLM applications in this field remain limited, focusing primarily on education, communication, and academic support, while the persistent risk of hallucinations underscores the need for professional oversight.^30^ Within this context, no AI-based chatbot specifically designed for postoperative care following tooth extractions has been identified in the literature, highlighting an important gap and reinforcing the relevance of the approach proposed in the present study.

Some limitations should be considered when interpreting the findings of this study. As with any LLM-based system, the chatbot’s performance depends on probabilistic text generation and may be affected by contextual misunderstandings or omissions, particularly when user input is ambiguous or incomplete. Although the rule-based framework constrains the model to postoperative guidance, it does not fully eliminate the inherent risks associated with LLM-generated responses, reinforcing the need for ongoing professional oversight to ensure safe clinical integration. The evaluation was conducted using simulated scenarios rather than real patient interactions, limiting the generalizability of the findings to clinical practice. In addition, the simulated scenarios primarily represented uncomplicated postoperative conditions and therefore may not fully reflect the complexity of routine clinical care. Future studies should incorporate more complex scenarios involving postoperative complications to further evaluate the system’s robustness and clinical applicability. Moreover, the binary evaluation approach assessed only the presence of essential information and did not capture qualitative aspects of the responses, including clarity, depth of explanation, and linguistic quality, all of which may influence patient understanding. Despite these limitations, NLP-based tools such as PostOPbot illustrate the potential of AI to support postoperative monitoring by providing structured, accessible, and time-efficient guidance for patients recovering from dental extractions. When implemented responsibly, such systems may improve patient understanding, promote adherence to postoperative recommendations, and broaden access to reliable information.

## Conclusion

In simulated scenarios, PostOPbot demonstrated consistent performance, providing most expected postoperative recommendations with few omissions and maintaining stable responses across different postoperative time points. When explicitly prompted, the system successfully generated recommendations that had initially been omitted. These findings suggest that the chatbot can provide standardized postoperative guidance and may serve as a complementary tool to support patients during the recovery period without replacing professional evaluation.

## Data Availability

The authors declare that all data generated or analyzed during this study are included in this published article.
